# Safety of Extended Propofol Infusions in Critically Ill Pediatric Patients

**DOI:** 10.7759/cureus.59948

**Published:** 2024-05-09

**Authors:** Daniel Moas, Elber Y Aydin, Jose Irazuzta, Stephanie Filipp, Kourtney K Guthrie, Kalen Manasco, Charlene Pringle

**Affiliations:** 1 Pediatric Critical Care Medicine, University of New Mexico School of Medicine, Albuquerque, USA; 2 Pediatrics, University of Florida College of Medicine-Jacksonville, Jacksonville, USA; 3 Pediatric Critical Care, University of Florida College of Medicine – Jacksonville, Jacksonville, USA; 4 Health Outcomes and Biomedical Informatics, University of Florida College of Medicine, Gainesville, USA; 5 Pediatrics, Shands Hospital at the University of Florida, Gainesville, USA; 6 Pharmacology and Therapeutics, University of Florida College of Medicine, Gainesville, USA; 7 Pediatrics, University of Florida College of Medicine, Gainesville, USA; 8 Critical Care Medicine, University of Florida College of Medicine, Gainesville, USA

**Keywords:** propofol-related infusion syndrome, rhabdomyolysis, acidosis, propofol, pediatric

## Abstract

Introduction

Propofol is a phenol agent with sedative and anesthetic properties that has been in use for decades, but with controversy in critically ill pediatric patients, given the concern for developing propofol-related infusion syndrome (PRIS). Our aim was to assess the risk of propofol infusions in the pediatric intensive care unit (PICU) at doses and durations greater than the described safety data and its associated covariables.

Methods

Retrospective cohort analysis of 173 patients receiving propofol in the PICU. Patients were categorized as receiving greater or less than 48-hour infusions. Demographic data and daily clinical variables were recorded for up to seven days post-infusion initiation or until infusion was stopped.

Results

In this descriptive analysis, patients’ demographics were similar, but admission diagnosis was not. Both groups received high mean doses of propofol (>67 mcg/kg/min), with no cases of PRIS observed. The illness severity scores and the need for vasoactive infusion support varied between the cohorts, with higher illness scores and a higher percentage of subjects requiring vasoactive agents in the >48-hour cohort. Finally, there were no major differences in lactate levels or biochemical characteristics between the two groups.

Conclusions

This study provides pilot data in relation to the feasibility of propofol infusion in critically ill pediatric patients and underscores the need for a larger multicenter study to draw clinical recommendations.

## Introduction

Propofol, or 2,6-diisopropylphenol, is a highly lipophilic alkylphenol hypnotic agent whose sedative effects were first identified in 1973 in mice [1]. Thirteen years of investigation and development resulted in approval for human use in Europe in 1986, followed by FDA approval in October 1989. Since then, propofol’s versatile characteristics, rapid onset and recovery times, and amnestic effects have led to its widespread use in many clinical areas. It is used to induce general anesthesia or as part of a regimen for total intravenous anesthesia [2]. It is also an excellent choice for procedural sedation, is frequently used for patients undergoing prolonged mechanical ventilation in intensive care units (ICUs), and is also used successfully in cases of refractory status epilepticus [2].

By 1992, propofol-related infusion syndrome (PRIS) was described in a case series of five children who died from cardiovascular collapse after long-term propofol infusions lasting 96-144 hours [3]. Clinical manifestations included hypotension, cardiac arrhythmias, myocardial failure, evidence of skeletal muscle breakdown, high anion gap acidosis from lactic acid, renal failure, hyperkalemia, hepatic transaminitis, hypertriglyceridemia, and hyperthermia [3-5]. Another case series by Bray et al. of 18 patients who developed PRIS receiving propofol in a PICU described all subjects receiving infusions >4 mg/kg/h (>67 mcg/kg/min) and lasting >48 hours [4]. The exact mechanism of PRIS has not been elucidated, but given its highly lipophilic nature and the clinical presentation, the evidence points toward mitochondrial toxicity from the decoupling of cellular respiration along the electron transport chain as well as disruption of fatty acid and acylcarnitine metabolism [6-8]. Given the suspected pathophysiology involved with PRIS, blood lactate levels are the most used marker of mitochondrial dysfunction in clinical settings, particularly when comparing them to previous levels in patients where there is concern for PRIS. As cellular respiration is disrupted, markers such as creatine kinase (CK), transaminase, and lipase are monitored to evaluate end-organ injury. Pyruvate is another biomarker that could be used, especially in the context of comparison to lactate levels. However, turnaround times for pyruvate can be very long and often need to be sent to specialty labs from many institutions. 

From these findings, a randomized controlled trial was initiated with 327 PICU patients which went unpublished and stopped early when a dose-dependent mortality relationship at 28 days was found [9,10]. That study reported 25 deaths at 28 days, 21 of which were receiving propofol [9]. Of those 21 subjects, 12 were receiving the 2% formulation which is not traditionally used in the United States, last in 2021 during an emergency use authorization by the FDA during a propofol 1% critical shortage [11]. Additionally, 10 of the 21 deaths at 28 days were recorded at one center. This center reported the use of higher doses of propofol, 2% formulation of propofol as opposed to the standard 1% formulation, higher doses of fentanyl, more use of total parenteral nutrition (TPN), and more patients with severe sepsis at baseline when compared to the other centers. When that center was excluded, mortality was similar between propofol and nonpropofol groups [9]. A subsequent 2001 FDA warning was published against the use of propofol for sedation in the PICU.

Since then, propofol’s use in pediatric critical care patients has been investigated frequently, however mostly in the context of affirming the safety parameters delineated by Bray et al. [4]. Several national surveys and single-center retrospective studies have corroborated the safety of propofol in critically ill children while observing the parameters of <4 mg/kg/h infusion rates and infusion durations of <48 hours in the ICU and mentioned the presence of metabolic diseases and mitochondrial disease as well as illness severity as possible risk factors [10,12-14]. Some studies suggest limiting its use to the operating room by trained anesthesiologists [15]. One common practice, with some evidence to support its use, is frequent laboratory monitoring of biochemical parameters to surveil for the development of PRIS [5,16]. However, there is evidence that such practice can falsely reassure practitioners as PRIS is a rapidly developing and theoretically irreversible phenomenon [17]. Finally, more data is emerging indicating doses temporarily above 4 mg/kg/h or infusions lasting longer than 48 hours might not definitively be the key determining risk factors for developing PRIS [18,19]. One single-center retrospective study of 174 children receiving propofol found doses of >3 mg/kg/h or durations of >48 hours to be associated with the development of metabolic or circulatory derangements [18], while another recent single-center retrospective study of 33 patients receiving propofol with a mean dose of 3 mg/kg/h and peaking at 4.5 mg/kg/h for at least eight hours, and in some cases >100 hours, similarly showed no significant metabolic or hemodynamic derangements [19].

The current state of propofol use in critically ill pediatric patients by center varies as widely as the literature. Some centers do not utilize the drug at all in their PICU. Some utilize the drug despite the FDA’s black box warning for pediatric use. Its use can be limited to following the safety parameters delineated by Bray et al., and others surpass the 4 mg/kg/h and 48-hour postulated limits [4]. Many, but not all centers, protocolize biochemical laboratory monitoring for patients receiving propofol for any amount of time in a PICU. Most recently, the 2022 PANDEM guidelines commented on the use of propofol in the PICU stating that use of propofol for less than 48 hours and at doses less than 4 mg/kg/h may be a safe sedation alternative in the PICU [16]. This recommendation was conditional and based on low-quality evidence. While there is safety data for lower-dose and duration propofol, the data showing increased morbidity and mortality with longer infusions or higher rates is limited to very small case series without control, and an unpublished study where appropriate propensity score matching for confounding variables was not described. Our aim was to assess the risk of propofol infusions in the PICU at doses and durations greater than the described safety data as well as its associated risk factors beyond dose and duration.

## Materials and methods

Design

We conducted a retrospective cohort pilot study in pediatric patients in the PICU who received propofol at the University of Florida’s Shand’s Children’s and Wolfson Children’s Hospitals. The study was determined by the hospitals' Institutional Review Boards to be exempt.

Patient selection

**Figure 1:**
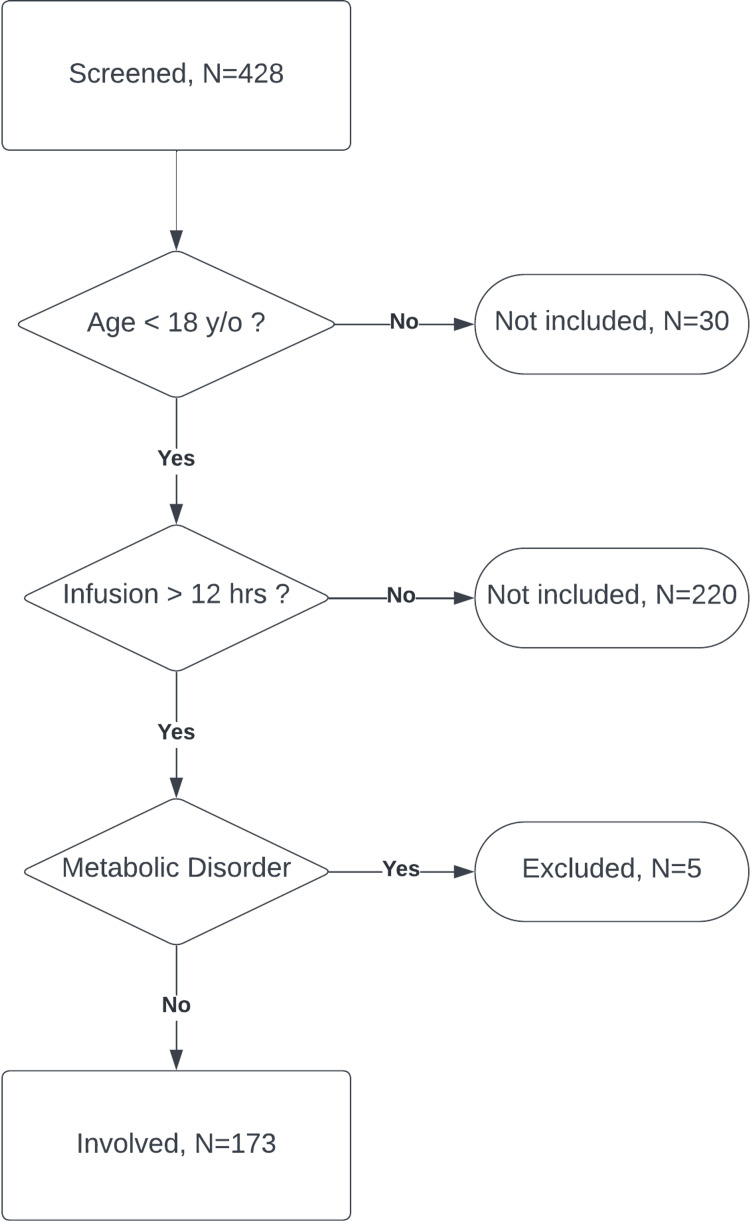
Patient selection

Subjects were identified by query of electronic health record (EHR) systems for all patients who received propofol for greater than 12 consecutive hours admitted to the PICU and pediatric cardiac ICU and trauma ICU between January 2017 to December 2021 and were of age 0 day to 17 years at admission. Patients with documented inborn errors of metabolism were excluded (Figure 1). Patient demographics are described in Table 1.

**Table 1:** Demographics TBI: Traumatic brain injury; WLST: withdrawal of life-sustaining therapy; LOS: length of stay; PICU: pediatric intensive care unit Covariates age, BMI, and LOS are displayed as median with interquartile range (IQR); otherwise, percentages are reported as n(%)

	Overall	≤48-hour infusion	>48-hour infusion
(n)	173	149 (86.1%)	24 (13.9%)
Site			
UF/Shands	109 (63.0%)	88 (59.1%)	21 (87.5%)
Wolfson	64 (37.0%)	61 (40.9%)	3 (12.5%)
Sex: male	108 (62.4%)	92 (61.7%)	16 (66.7%)
Age (years)	8.0 (2.0, 14.0)	6.0 (2.0, 13.0)	13.5 (8.0, 16.0)
BMI	18.4 (16.0, 22.0)	18.0 (15.9, 21.9)	19.3 (18.3, 25.5)
Race/ethnicity			
Non-Hispanic White	96 (55.5%)	85 (57.1%)	11 (45.8%)
Non-Hispanic Black	47 (27.2%)	39 (26.2%)	8 (33.3%)
Hispanic	17 (9.8%)	12 (8.1%)	5 (20.8%)
Other	13 (7.5%)	13 (8.7%)	--
Washout for extubation: yes	48 (27.8%)	46 (30.9%)	2 (8.3%)
Admitting diagnosis			
TBI	24 (13.9%)	19 (12.8%)	5 (20.8%)
Burn	6 (3.5%)	4 (2.7%)	2 (8.3%)
Drowning	2 (1.2%)	2 (1.3%)	--
Acute respiratory failure	23 (13.3%)	20 (13.4%)	3 (12.5%)
Sepsis	4 (2.3%)	3 (2.0%)	1 (4.2%)
Postsurgical procedure	47 (27.2%)	44 (29.5%)	3 (12.5%)
Status epilepticus	27 (15.6%)	25 (16.8%)	2 (8.3%)
Out-of-hospital cardiac arrest	3 (1.7%)	2 (1.3%)	1 (4.2%)
Hypoxic-ischemic brain injury	4 (2.3%)	3 (2.0%)	1 (4.2%)
Other trauma	6 (3.5%)	6 (4.0%)	--
Other	27 (15.6%)	21 (14.1%)	6 (25.0%)
Inpatient death: yes	24 (13.9%)	20 (13.4%)	4 (16.7%)
Cause of death (n = 24):			
WLST	12 (50.0%)	9 (45.0%)	1 (25.0%)
Admit diagnosis	9 (37.5%)	8 (40.0%)	3 (75.0%)
Other complication(s)	3 (12.5%)	3 (15.0%)	--
28-day mortality	18 (10.4%)	16 (10.7%)	2 (8.3%)
LOS hospital (days)	10.0 (4.0, 28.0)	8.0 (3.0, 23.0)	33.0 (20.0, 67.0)
LOS PICU (days)	8.0 (3.0, 24.0)	6.0 (3.0, 17.0)	32.0 (20.0, 67.0)

Data collection

Data gathered from subject’s electronic charts included demographics, illness severity scores (pediatric logistic organ dysfunction-2 (PELOD-2) and pediatric sequential organ failure assessment (pSOFA)), mean and max propofol infusion doses per day, total daily propofol administered per study day, propofol infusion duration per study day, incidence of dysrhythmias, cardiac arrest, and the need for extracorporeal life support (ECLS) (extracorporeal membrane oxygenation (ECMO), continuous renal replacement therapy (CRRT) or ventricular assist device (VADs)), vasoactive index score per study day, incidence of rhabdomyolysis, peak lactate, creatine kinase, triglyceride, alanine and aspartate aminotransferase and lipase levels per study day, and the in-hospital and 28-day mortality. It was noted that propofol indication was for pre-extubation sedation “washout” per each subject. For each subject, all admissions within the study period were screened for propofol infusion in an ICU lasting longer than 12 hours. If a subject had more than one admission that met the criteria, each admission was a separate subject encounter that was recorded. Within each admission for a subject, the longest continuous dose of propofol in the ICU was the study period for that admission. The end of a discrete infusion period was considered when the infusion was stopped for more than one hour. Pauses less than one hour, such as those for neurologic checks, were disregarded, and the infusion was considered continuous. For each subject’s study period, the day of propofol initiation was termed study day 0, and data was abstracted up to study day 7 for each subject or until the infusion was discontinued if less than seven days.

Statistical analysis

Continuous data was all skewed, non-normally distributed data and as such were reported as median (25th and 75th percentiles). Categorical data was reported as frequency and percentages. Fisher’s exact test was used for all admitting diagnosis, binary indicators, and inpatient and 28-day mortality. Chi-squared test was used for sex, data collection site, and “washout” indication. T-tests were used for continuous data except for those with unequal variances, where Satterthwaite test was used. Given the heavily skewed cohort groups and inadequate sample size, descriptive analysis was performed in lieu of the reporting of formal statistical analysis. Lastly, study days 0 and 1 reported and analyzed, as study days 2-7 had limited data collected, with study day 2 only having 21 subjects with reported data in the <48-hour group (Table 2).

**Table 2:** Daily rate of those remaining in the study within each group Data displayed as n(%)

	Study day 0	Study day 1	Study day 2	Study day 3	Study day 4	Study day 5
≤48-hour infusion group (mcg/kg/min)	149 (100.0%)	109 (73.2%)	21 (14.1%)	0 (0.0%)	0 (0.0%)	0 (0.0%)
>48-hour infusion group (mcg/kg/min)	24 (100.0%)	24 (100.0%)	23 (95.8%)	13 (54.2%)	5 (20.8%)	3 (12.5%)

## Results

Patient characteristics

A total of 173 subjects were enrolled between both hospitals, 149 (86.1) in the <48-hour cohort and 24 (13.9) in the >48-hour cohort. Overall subjects were 62.4% male, with 92 (61.7) in the <48-hour group and 16 (66.7) in the >48-hour group. Patients in the >48-hour group tended to be older with 13.5 (8.0, 16.0) compared to those in the <48-hour group with 6.0 (2.0, 13.0) with an overall median age of 8 (2.0, 14.0) among all patients. The median BMI for the study subjects was 18.4 (16.0, 22.0), with the <48-hour group having a median of 18.0 (15.9, 21.9) and the >48-hour group, 19.3 (18.3, 25.5). Overall, no differences in race/ethnicity were noted between the cohorts. The <48-hour cohort had substantially more subjects whose indication for propofol was recorded as “washout” prior to extubation as one would expect. While admitting diagnoses frequently varied between groups (Table 1), this can be clearly attributed to the small sample size of the >48-hour cohort. Lastly, mortality was not strikingly different, with overall in-hospital mortality being 24 (13.9). There were 20 (13.4) subjects in the <48-hour cohort and 4 (16.7) in the >48-hour group. There was a similar comparison for the 28-day mortality, overall being 18 (10.4) and 16 (10.7) in the <48-hour group and 2 (8.3) in the >48-hour group.

Clinical characteristics

No diagnosis of PRIS was made in either group. Both groups received what would be considered high doses of propofol at mean doses of >67 mcg/kg/min (4 mg/kg/h), with the <48-hour cohort receiving 83 mcg/kg/min (55.0, 108.3) and the >48-hour cohort receiving 74.9 mcg/kg/min (49.5, 89.4) on study day 0 (Table 3). A similar comparison for study day 1 demonstrates the <48-hour cohort receiving 98 mcg/kg/min (63.0, 126.3) and the >48-hour cohort receiving 70.6 mcg/kg/min (50.0, 113.0) on average (Table 4). Illness severity scores seem to have varied slightly, with the >48-hour cohort having slightly higher scores. PELOD-2 scores for the >48-hour cohort on study days 0 and 1 being 6.0 (4.0, 8.5) and 6.5 (5.0, 9.0), respectively, compared to the <48-hour group being 4.0 (3.0, 6.0) on both days. For pSOFA scores, the >48-hour group were once again slightly higher, with values being 5.0 (2.0, 7.0) and 5.0 (3.0, 9.5) on study days 0 and 1, respectively, compared to the <48-hour group’s values being 3.0 (2.0, 5.0) and 3.0 (2.0, 4.0) on days 0 and 1, respectively (Tables 3-4).

**Table 3:** Study day 0 characteristics ECLS: Extracorporeal life support; ECMO: extracorporeal membrane oxygenation; CRRT: continuous renal replacement therapy; PELOD-2: pediatric logistic organ dysfunction-2; pSOFA: pediatric sequential organ failure assessment; VIS: vasoactive-inotropic score; PRIS: propofol-related infusion syndrome Study days are displayed as mean +/- SD. Clinical characteristics are displayed as n(%). All other covariates are displayed as median with interquartile range (IQR)

	(n)	Overall	(n)	≤48-hour infusion	(n)	>48-hour infusion
# of study days	173	2.1 ± 1.0	149	1.9 ± 0.6	24	3.8 ± 1.1
Propofol						
Total hours of propofol infusion	173	12.0 (8.0, 16.0)	149	12.0 (8.0, 15.0)	24	12.0 (7.0, 16.0)
Propofol requirement (mg/kg)	173	48.0 (27.0, 79.6)	149	50.0 (27.0, 82.0)	24	43.1 (28.8, 73.6)
Max single-dose propofol (mcg/kg/min)	173	100.0 (75.0, 150)	149	100.0 (75.0, 150.0)	24	100.0 (60.0, 125.0)
Mean propofol dose (mcg/kg/min)	173	79.0 (52.0, 103.5)	149	83.0 (55.0, 108.3)	24	74.9 (49.5, 89.4)
Clinical characteristics						
Dysrhythmia	173	4 (2.3%)	173	4 (2.7%)	24	0 (0.0%)
Cardiac arrest	173	0 (0.0%)	173	0 (0.0%)	24	0 (0.0%)
ECLS	173	6 (3.5%)	173	5 (3.4%)	24	1 (4.2%)
ECLS present prior day	6	6 (100.0%)	5	5 (100.0%)	1	1 (1.00%)
Type of ECLS	6		5		1	
ECMO		3 (50.0%)		2 (40.0%)		1 (100.0%)
CRRT		2 (33.3%)		2 (40.0%)		0 (0.0%)
Other		2 (33.3%)		2 (40.0%)		0 (0.0%)
PELOD-2 score	173	4.0 (3.0, 6.0)	149	4.0 (3.0, 6.0)	24	6.0 (4.0, 8.5)
pSOFA score	173	3.0 (2.0, 6.0)	149	3.0 (2.0, 5.0)	24	5.0 (2.0, 7.0)
Catecholamine infusion	173	35 (20.2%)	149	26 (17.5%)	24	9 (37.5%)
(Among yes) VIS score	35	7.0 (3.0, 15.0)	26	5.0 (3.0, 15.0)	9	7.0 (5.0, 15.0)
PRIS diagnosis	173	0 (0.0%)	149	0 (0.0%)	24	0 (0.0%)
Rhabdomyolysis	173	1 (0.6%)	149	1 (0.7%)	24	0 (0.0%)
Bloodwork						
Serum lactate (mmol/L) (highest)	121	1.5 (1.0, 2.6)	99	1.5 (1.0, 2.8)	22	1.7 (1.2, 2.2)
Serum creatinine kinase (U/L) (highest)	14	282.0 (126.0, 648.0)	11	319.0 (216.0, 1516.0)	3	143.0 (99.0, 648.0)
Serum AST (U/L) (highest)	101	42.0 (24.0, 81.0)	88	41.5 (24.0, 82.0)	13	45.0 (25.0, 79.0)
Serum ALT (U/L) (highest)	100	21.5 (13.5, 48.0)	87	20.0 (14.0, 44.0)	13	25.0 (13.0, 77.0)
Serum Lipase (U/L) (highest)	22	16.0 (10.0, 51.0)	17	17.0 (10.0, 36.0)	5	10.0 (10.0, 95.0)

**Table 4:** Study day 1 characteristics ECLS: Extracorporeal life support; ECMO: extracorporeal membrane oxygenation; CRRT: continuous renal replacement therapy; PELOD-2: pediatric logistic organ dysfunction-2; pSOFA: pediatric sequential organ failure assessment; VIS: vasoactive-inotropic score; PRIS: propofol-related infusion syndrome Clinical characteristics are displayed as n(%). All other covariates are displayed as median with interquartile range (IQR)

	(n)	Overall	(n)	≤48-hour infusion	(n)	>48-hour infusion
	133		109		24	
Propofol						
Total hours of propofol infusion	133	14.0 (11.0, 24.0)	109	12.0 (10.0, 17.0)	24	24.0 (24.0, 24.0)
Propofol requirement (mg/kg)	133	72.0 (45.0, 108.0)	109	67.0 (44.6, 105.9)	24	93.2 (72.0, 162.8)
Max single-dose propofol (mcg/kg/min)	133	100.0 (75.0, 150.0)	109	100.0 (75.0, 150.0)	24	100.0 (75.0, 150.0)
Mean propofol dose (mcg/kg/min)	133	91.6 (62.3, 126.0)	109	98.0 (63.0, 126.3)	24	70.6 (50.0, 113.0)
Clinical characteristics						
Dysrhythmia	133	2 (1.5%)	109	2 (1.8%)	24	0 (0.0%)
Cardiac arrest	133	2 (1.5%)	109	2 (1.8%)	24	0 (0.0%)
ECLS	133	4 (3.0%)	109	3 (2.8%)	24	1 (4.2%)
ECLS present prior day	4	4 (100.0%)	3	3 (100.0%)	1	1 (100.0%)
Type of ECLS	4		3		1	
ECMO		3 (75.0%)		2 (66.7%)		1 (100.0%)
CRRT		2 (50.0%)		2 (66.7%)		0 (0.0%)
Other		0 (0.0%)		0 (0.0%)		0 (0.0%)
PELOD-2 score	132	4.0 (3.0, 6.0)	108	4.0 (3.0, 6.0)	24	6.5 (5.0, 9.0)
pSOFA score	132	3.0 (2.0, 5.0)	108	3.0 (2.0, 4.0)	24	5.0 (3.0, 9.5)
Catecholamine infusion	132	26 (19.7%)	108	16 (14.8%)	24	10 (41.7%)
(Among yes) VIS score	25	10.0 (4.0, 18.0)	16	8.5 (3.5, 19.0)	9	10.0 (7.0, 14.0)
PRIS diagnosis	132	0 (0.0%)	108	0 (0.0%)	24	0 (0.0%)
Rhabdomyolysis	132	1 (0.8%)	108	1 (0.9%)	24	0 (0.0%)
Bloodwork						
Serum lactate (mmol/L) (highest)	76	1.3 (0.9, 2.0)	55	1.2 (0.8, 2.0)	21	1.6 (1.2, 2.0)
Serum creatinine kinase (U/L) (highest)	13	444.0 (142.0, 692.0)	9	444.0 (295.0, 1247.0)	4	362.0 (85.5, 609.0)
Serum AST (U/L) (highest)	46	52.5 (25.0, 90.0)	37	46.0 (25.0, 73.0)	9	72.0 (55.0, 109.0)
Serum ALT (U/L) (highest)	46	39.5 (16.0, 102.0)	37	38.0 (13.0, 90.0)	9	88.0 (26.0, 105.0)
Serum lipase (U/L) (highest)	6	19.0 (13.0, 285.0)	5	16.0 (13.0, 22.0)	1	N/A

The need for vasoactive infusion support varied between the two groups, with 37.5% and 41.7% of subjects from the >48-hour cohort requiring vasoactive agents on days 0 and 1, respectively, compared to 17.5% and 14.8%, respectively, for the <48-hour cohort. Regarding the amount of vasoactive support needed, vasoactive index scores were similar between both cohorts with study days 0 and 1 for the >48-hour cohort being 7.0 (5.0, 15.0) and 10.0 (7.0, 14.0), respectively, while the <48-hour group scored 5.0 (3.0, 15.0) and 8.5 (3.5, 19.0), respectively (Tables 3-4). There were no cardiac arrests in either group on study day 0. The <48-hour cohort had two cardiac arrests recorded on study day 1. The incidence of dysrhythmias was higher in the larger <48-hour cohort, with four on study day 0 and two on study day 1, while the >48-hour cohort had none (Tables 3-4), which is clearly affected by the small sample size of the >48-hour cohort. ECLS was implemented within both groups at low incidences, with all implementations occurring prior to initiation of propofol infusion.

Biochemical characteristics

The lactate levels of the two groups did not seem to differ. The <48-hour cohort recorded lactate levels of 1.5 (1.0, 2.8) and 1.2 (0.8, 2.0) on study days 0 and 1, while the >48-hour cohort recorded levels of 1.7 (1.2, 2.2) and 1.6 (1.2, 2.0) on days 0 and 1 (Tables 3-4). Serum creatine kinase did not seem to differ greatly, but few subjects had these values tested making even a descriptive comparison impossible. Serum lipase and triglycerides were rarely tested, sometimes not at all, similarly making a comparison impossible.

## Discussion

While there have been similar studies, most recently Uppuluri et al., this is the first study of this size to attempt to collect and report a complete clinical and biochemical profile of critically ill pediatric patients receiving propofol infusions of varying dose and duration [19]. While this study serves as a small pilot study with the intention of a larger multicenter study to be able to adequately power robust statistical comparisons, some preliminary descriptive comparisons of the data set are of interest. As described, there was no observed difference in mortality between the two groups when comparing all study days. Both groups received similar mean and peak doses of propofol on the two study days reported (days 0 and 1). There was clearly no capability to assess the confounding effects of any suspected clinical variables potentially contributing to mortality. Particularly interesting are the BMI, admitting diagnosis, illness severity score, use of vasoactive medications, and use of steroids [10].

As far as a comparison of the two cohorts in this data set, BMI and race/ethnicity did not overtly differ. The admission diagnosis varied wildly between groups, but these differences are ones that are not completely unexpected. For example, patients with traumatic brain injury (TBI) are frequently maintained on propofol at the participating institutions to facilitate frequent neurologic exams. Status epilepticus and simple surgeries are admission indications that frequently require less than 48 hours of mechanical ventilation and lead to a practitioner’s reassurance of propofol use. The data collected seems to reflect the natural history of the patient’s diagnosis and the support they require.

The comparison of the two cohorts for the first two study days carries little meaning, as they are effectively both data sets reflecting 48 hours of propofol or less. It is, however, interesting to note that early on, the >48-hour cohort had some slight but important differences emerging. There was considerably more usage of vasoactive agents in the >48-hour group noted even at study day 0. Additionally, PELOD-2 and pSOFA scores were slightly higher in that cohort compared to the <48-hour group. Clearly whether these differences would be statistically significant in a well-sized sample population needs to be determined.

Certain diagnoses lend themselves to longer need for mechanical ventilation and sedation and that these might be processes that can either benefit directly from propofol’s hemodynamic side effect profile or from its favorable pharmacokinetics allowing for rapid titrations and clinical assessments when needed. It is not a stretch to imagine that some of these diagnoses are ones that at baseline carry a greater risk of morbidity and mortality and that might confound case series reporting increased mortality associated with prolonged or high-dose propofol infusions [3,4].

PRIS has clearly been described with enough consistency of clinical and biochemical presentation to understand that the use of propofol in critically ill children has real risks [3,4,12,15]. The question remains which patients are at greatest risk and what are the pharmacologic, demographic, and clinic variables that need to be accounted for and considered when selecting which patients are or are not appropriate to receive propofol infusions. While there is reasonable data to support its cautious use in many patients while maintaining infusion rate at <4 mg/kg/h and duration less than 48-hour, propofol itself can be a tremendous asset in certain clinical scenarios outside of those parameters, and delineating which patients are likely to tolerate higher doses or longer durations and remain safe can be of tremendous benefit to both patients and practitioners.

Our study has its limitations. Given propofol’s judicious use among institutions, two centers do not represent a sample size to adequately power meaningful statistical comparisons. To obtain an adequate sample population a large multicenter prospective cohort study would be needed. Another aspect to consider is the absence of any patients who developed PRIS, which poses another constraint on our ability to analyze the characteristics that could potentially predispose individuals to this condition. This study certainly can serve as a template for such a study and the incipiency of a much-needed data set. The retrospective nature of the study is a limitation, as the population studied is heterogeneous and direct comparisons are difficult, particularly when the sample population is small. Similarly, the retrospective nature limits the capture of the desired data, such as certain laboratory values.

## Conclusions

Our study describes the pilot data for an intended large multicenter study following the above protocol. While clearly no conclusions can be drawn from the described data, further questions regarding the contributory risk factors to develop PRIS have been highlighted and should be clearly answered with a study of a proper sample population. Should certain variables become suspect risk factors from a larger multicenter retrospective study, prospective controlled clinical trials for the populations of interest can be undertaken.
